# Combination of Platelet-Rich Fibrin and Carbonate Hydroxyapatite for Bone Regeneration in the Management of Radicular Cyst: A Case Report

**DOI:** 10.1155/crid/3254011

**Published:** 2025-09-21

**Authors:** Muhammad Irfan Rasul, Kasma AS, Nurhayaty Natsir

**Affiliations:** ^1^Department of Oral and Maxillofacial Surgery, Faculty of Dentistry, Hasanuddin University, Makassar, Indonesia; ^2^Hasanuddin University Dental Hospital, Makassar, Indonesia; ^3^Department of Conservative Dentistry and Endodontics, Faculty of Dentistry, Hasanuddin University, Makassar, Indonesia

**Keywords:** bone regeneration, carbonate hydroxyapatite, platelet-rich fibrin, radicular cyst

## Abstract

**Introduction:** Radicular cysts are the most common type of inflammatory cyst of the jaw. Management of radicular cysts may include cyst enucleation with apical resection. Spontaneous bone healing after cyst enucleation has been reported to occur in 73.5% of cases after 12 months. Residual cavity size was reduced 12.34% after 6 months, 43.46% after 12 months, and 81.30% after 24 months after surgery. This case report presents the use of platelet-rich fibrin and carbonate hydroxyapatite in the management of a radicular cyst for bone regeneration. This combination has not been previously reported.

**Case Report:** A 31-year-old male complained of swelling of the anterior maxilla for 1.5 years. The patient had a history of pain and previous dental restorations. A biopsy confirmed the diagnosis of a radicular cyst. The cyst in the tooth region 21–23 was treated with enucleation of the cyst and apical resection. We used PRF with CHA on the cyst defect to enhance healing and bone regeneration. CBCT showed that bone density increased, and the size defect area buccal–palatal expansion reduced 54.4%, and mesial–distal expansion reduced 35.7% after 6 months of surgery.

**Conclusion:** PRF and CHA may be used to accelerate bone regeneration in radicular cyst management.

## 1. Introduction

Radicular cysts are the most common inflammatory cysts, accounting for 52%–68% of all jaw cysts. Radicular cysts arise due to the proliferation of epithelial remnants in the periodontal ligament due to periapical periodontitis, followed by pulp necrosis. Periapical inflammation stimulates epithelial cell rests of Malassez in the apical periodontal ligament to form a periapical granuloma. Then, this epithelium necrosis is due to a lack of blood supply, and the granuloma turns into a cyst called a radicular cyst [1–3]. The treatment of radicular cysts can be performed nonsurgically or by surgical methods such as marsupialization and enucleation, depending on the size and location of the cyst, the bone integrity of the cystic wall, and proximity to vital structures [4]. Cyst enucleation is a radical method to remove all of the cyst capsule [5]. Apical resection is a surgical technique that involves removing the tip of the tooth root and closing the apical portion of the root canal. Apical resection is aimed at removing pathological periapical tissue from the root surface, providing a blockage so that endodontic treatment does not leak, and creating conditions suitable for the physiological recovery of apical tissues [6].

Large cysts greater than 3 cc had larger defects. These large cyst defects carry increased postoperative risks, such as pathologic fracture, limited mouth opening, and inadequate bone healing. Residual cavity size was reduced 12.34% after 6 months, 43.46% after 12 months, and 81.30% after 24 months after surgery [7, 8]. Kui et al. in their study concluded that the spontaneous bone healing ratio of defects was 73.5% after 12 months of enucleation. Additional treatment, such as bone grafting, may be considered after cyst enucleation [9].

Platelet-rich fibrin (PRF) was first introduced by Choukroun et al. in 2001. PRF is an immune and platelet concentrate collected on a single fibrin membrane containing all blood constituents favorable for healing and immunity. PRF releases through degranulation at least seven different growth factors and cytokines that stimulate bone and soft tissue healing. Thus, PRF is an easy and cost-effective way to obtain high concentrations of growth factors for soft and hard tissue regeneration in wound healing [10]. Bone grafts and bone regeneration materials are being used with varying degrees of success [11]. Carbonate hydroxyapatite (CHA) is a major inorganic component of natural bone. CHA has been used extensively in biomedical implant and bone regeneration applications because of its bioactive, biodegradable, and osteoconductive properties. CHA is also biocompatible, nontoxic, noninflammatory, and nonimmunogenic and can form a direct chemical bond with surrounding hard tissues. CHA is synthesized from calcium nitrate tetrahydrate, diammonium hydrogen phosphate, and sodium hydrogen carbonate. CHA is also more osteoconductive and more resorbable than hydroxyapatite (HA). CHA contains 3%–5% carbonate ions by substitution in the HA lattice structure and is the primary mineral constituent of the bone [12].

This case report presents the use of PRF and CHA in the management of a radicular cyst for bone regeneration. This combination has not been previously reported.

## 2. Case Report

A 31-year-old male patient complained of enlargement of the front maxillary region that had been experienced for approximately 1.5 years. There was a history of pain in the front teeth that had been filled about 2.5 years ago. There is no family history of the same condition. The patient wanted the cyst and the causative tooth treated.

**Figure 1:**
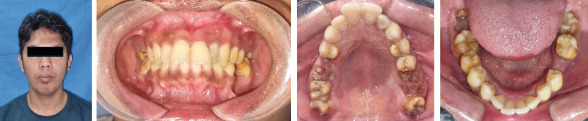
Extraoral and intraoral clinical features.

Extraoral examination found no abnormalities. On intraoral examination, there was an enlargement in the vestibule area of teeth 21–23, measuring ±1.5 0.8 × 0.5-cm size, localized, with soft consistency, palpation on pain (−), crepitus (+). There was enlargement of the vestibule of Teeth 46–47, measuring ±1 × 0.5 × 0.5-cm size, localized, with soft consistency, palpation on pain (−), crepitation (+), and deep caries of Tooth 22. Edentulous teeth 35 and 36 can be seen in Figure 1.

**Figure 2:**
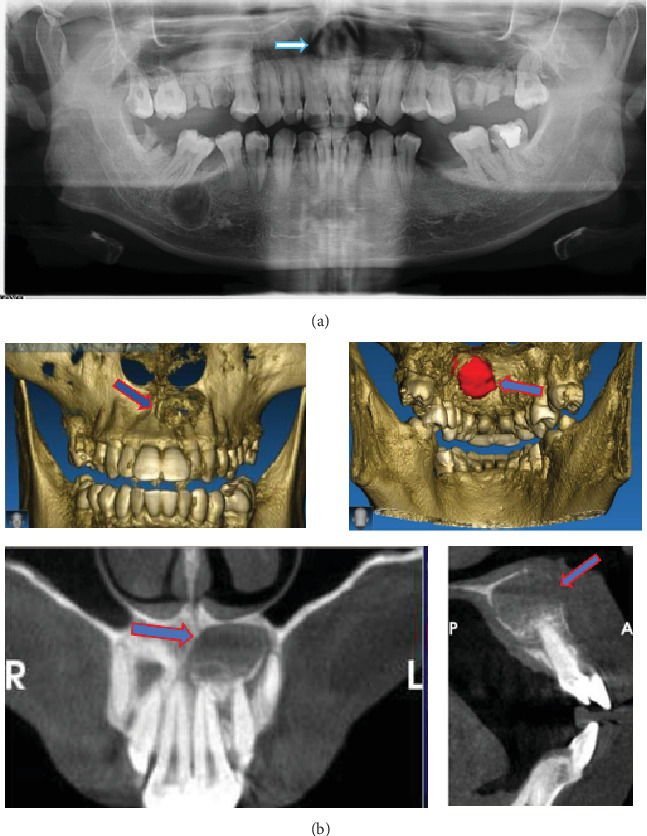
(a) Orthopantomogram X-ray feature. (b) CBCT of the anterior maxilla. The arrows indicate the location of the cyst area.

Radiological examination was performed with orthopantomography X-ray (OPG X-ray) and cone beam computed tomography (CBCT), with the impression of a suspected long-standing cystic lesion. Evidence of internal calcification (suspected radicular cyst and infected radicular cyst) can be seen in Figure 2.

**Figure 3:**
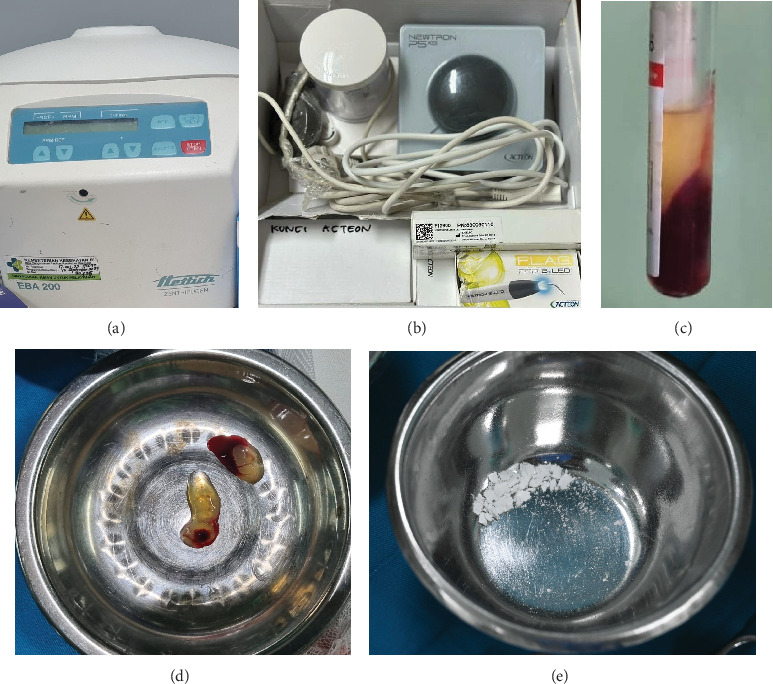
Preparation of tools and materials. (a) Centrifuge to obtain PRF. (b) Apex locator for apical resection. (c) Blood after centrifugation. (d) PRF. (e) CHA bone graft.

**Figure 4:**
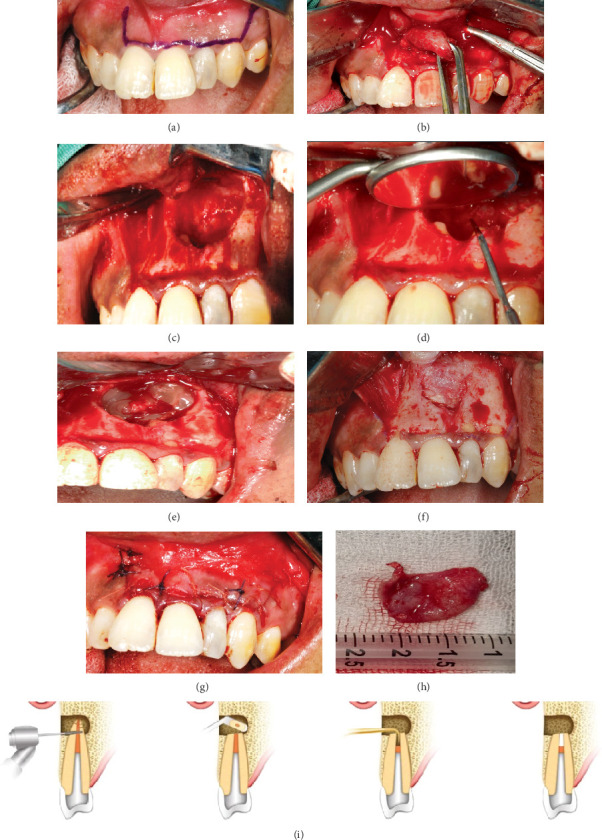
Procedures. (a) Marking of the surgical site. (b) Incision and mucoperiosteal flap of the surgical site and removal of the cyst sac. (c) Bone defect after cyst enucleation. (d) Apical resection of tooth 22, the cyst-causing tooth, was performed. (e) PRF and CHA were applied to the bone defect. (f) Closure of the bone defect using a membrane. (g) Suturing of the surgical site, (h) Result of surgery. (i) Illustrations of the apical resection procedure performed from the lateral view cited by Wang et al. [12].

**Figure 5:**
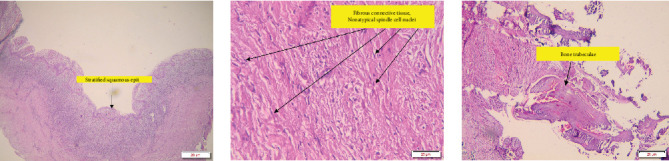
Microscopic features of histopathological examination showed a cystic structure lined with stratified squamous epithelium with atypical nuclei. The underlying stroma had a foamy distribution of macrophages and inflammatory cells, including lymphocytes, histiocytes, plasma cells, and some eosinophils. The conclusion of the histopathological examination was a radicular cyst.

The patient was diagnosed with a radicular cyst in the 21–23 tooth region. A history of a biopsy incision was performed, with the results supporting a radicular cyst. Root canal retreatment was performed on teeth 21–23 before apical resection. A combination of cyst enucleation with PRF + CHA and apical resection was performed on region Tooth 21–23, and cyst enucleation with PRF on region Tooth 47 under general anesthesia. The preparation of materials used can be seen in Figure 3. The procedure and illustrations of the lateral view apical resection [13] performed can be seen in Figure 4. The patient was given medicated ceftriaxone injection every 12 h, ketorolac injection every 8 h, ranitidine injection every 12 h, and dexamethasone injection every 12 h while in hospitalization. Histopathological examination results can be seen in Figure 5.

**Figure 6:**
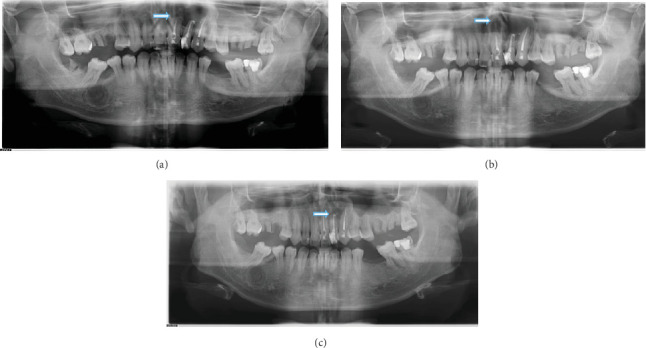
(a) Follow-up OPG X-ray 1 month after surgery showed a radiolucent area in the cyst defect decreased from the preoperative cyst size. (b) Follow-up OPG X-ray 3 months after surgery showed a radiolucent area in the cyst defect, smaller than the OPG X-ray 1 month after surgery. (c) Follow-up OPG X-ray 6 months after surgery showed a radiolucent area in the cyst defect getting smaller.

**Figure 7:**
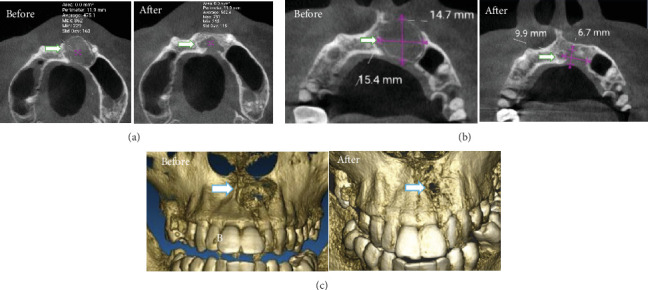
CBCT comparison before and 6 months after surgery. (a) X-ray mode from axial view shows bone density increased 6 months after surgery. (b) The size of the defect area, buccal–palatal expansion, and mesial–distal expansion from axial view reduces after surgery. (c) Bone model shows the cyst defect is still slightly present in Tooth 22, but the surrounding area shows there has been bone regeneration.

The patient was followed up 1 month, 3 months, and 6 months after surgery to see the patient's bone condition. OPG X-ray showed that the size of the radiolucent area in the cyst defect decreased compared to the preoperative cyst defect, as shown in Figure 6. Bone formation was measured using the AIS 3D Application (de Götzen, ACTEON Group). We compared CBCT images before surgery and 6 months after surgery, which showed that X-ray mode from axial view shows that bone density increased 6 months after surgery. The size defect area buccal–palatal expansion reduced 54.4%, and mesial–distal expansion reduced 35.7% after 6 months of surgery. Bone mode shows the cyst defect is still slightly present in Tooth 22, but the surrounding area shows there has been bone regeneration, as shown in Figure 7. At the 6-month follow-up after surgery, the patient did not complain of pain and swelling at the surgical site.

## 3. Discussion

Radicular cysts, also called periapical cysts or infected dental cysts, arise from the chronic inflammatory stimulation of epithelial cell rests of Malassez in the periodontal ligament [14]. They are the most common odontogenic cyst, affecting the maxilla more than three times the mandible. The pathogenesis process of a cyst begins with initiation, which gradually progresses to cyst formation and then enlarges to involve the adjacent bone and other vital structures in its surroundings [15]. Enucleation, so far, has been the most effective and reliable method to treat cysts. It completely removes the cystic capsule, therefore reducing the possibility of recurrence [16]. Enucleation cyst is the chosen treatment plan whenever the cyst is small, and saving the involved tooth is impossible [5]. Apical resection is considered the last resort to preserve natural teeth after the failure of endodontic treatment. The main goal of apical resection is to create a tight seal in the root apex, thereby preventing a pathway between the root canal system and peri-radicular tissues. Apical resection is a way to preserve the tooth causing the radicular cyst, so the patient does not lose the tooth [17].

This study was managed by enucleation and apical resection. In advance, we use PRF with CHA. Choukroun et al. (2001) developed a method of collecting platelets; the protocol aimed at collecting platelets and releasing cytokines in a fibrin clot. The fibrin matrix is the key for this product, as it supports cells during the initial healing phase [10]. The proteins derived from platelets include platelet-derived growth factor (PDGF), transforming growth factor (TGF-*β*), vascular endothelial growth factor (VEGF), and epidermal growth factor (EGF). Plasma contains certain natural growth factors, including insulin-like growth factor (IGF) and hepatocyte growth factor (HGF). The ability of PRGF to accelerate soft and hard tissue healing has stimulated the research of its clinical applications [10]. Normally, after the periapical surgery, approximately 1 year is required for complete healing. In contrast, with PRF, healing occurs fast, and complete regeneration of bone takes place in approximately 6 months. Thus, the application of autologous PRF as a surgical adjuvant yields new possibilities for enhanced healing and fast, functional recovery [18].

CHA is a calcium phosphate biomaterial for bone substitute that is biocompatible, bioactive, and osteoconductive [19]. CHA forms geometries well in various sizes and forms of mineralized bone. CHA can fill bone defects or irregular cavities. Increased carbonate substitution in CHA in bone formation depends on the function of osteoclasts [20]. CHA also tends to induce differentiation of spontaneous bone formation [21].

Elabdin et al. found that combining autogenous bone graft with PRF speeds up bone regeneration. They showed consistent clinical and radiographic evidence of faster bone formation and healing compared to using only PRF or not using grafting materials [11].

PRF and CHA work together to promote bone regeneration by offering a scaffold for cell growth and delivering growth factors. PRF, a blood-derived biomaterial, functions as a natural reservoir of growth factors and a physical barrier, while CHA supplies mineral components necessary for bone formation. The limitation of this study was located in the sample we used, which was one patient, but we committed to doing future research with a larger sample and long-term follow-up that will support this study.

## 4. Conclusion

A combination of PRF and CHA in managing radicular cysts can accelerate bone regeneration, as shown by an increase in bone density in the defect area after 6 months of surgery.

## Data Availability

The data that support the findings of this study are available from the corresponding author upon reasonable request.
